# CLDN18.2 in colorectal mucinous adenocarcinoma: a lineage-associated biomarker and emerging translational target

**DOI:** 10.3389/fphar.2026.2001935

**Published:** 2026-10-22

**Authors:** Wei Yu, Shengyu Wei, Shiqin Wan, Yiming Chen, Qiao Huang

**Affiliations:** 1 Department of Oncology, Affiliated Central People’s Hospital, China Three Gorges University, Yichang, Hubei, China; 2 Yichang Central People’s Hospital, Yichang, Hubei, China; 3 Tumor Prevention and Treatment Center of Three Gorges University and Cancer Research Institute of Three Gorges University, Yichang, Hubei, China; 4 Department of Gynecology, Affiliated Central People’s Hospital, China Three Gorges University, Yichang, Hubei, China

**Keywords:** biomarker, CLDN18.2, colorectal mucinous adenocarcinoma, companion diagnostics, targeted therapy

## Abstract

Colorectal mucinous adenocarcinoma (CMA) is a histologically and molecularly distinct subtype of colorectal cancer (CRC), marked by abundant extracellular mucin, a predilection for the proximal colon, and frequent overlap with deficient mismatch repair/high microsatellite instability (dMMR/MSI-H), BRAF V600E mutation, CpG island methylator phenotype-high (CIMP-high) status, and serrated pathway biology. Although immune checkpoint inhibitors and molecularly targeted therapies have expanded treatment options for selected patients with advanced CRC, treatment strategies tailored to the histologic phenotype, lineage programs, and microenvironmental architecture of CMA remain limited. CLDN18.2, the gastric isoform encoded by CLDN18, is now a clinically validated therapeutic target in gastric and gastroesophageal junction (GEJ) adenocarcinomas. Recent pathology-based studies suggest that CLDN18.2 expression is uncommon in unselected conventional colorectal adenocarcinoma but enriched in CMA, mucin-rich CRC, and tumors with gastric-type or foregut-like differentiation, particularly those arising in the proximal colon or harboring dMMR/MSI-H status or BRAF V600E mutation. This pattern suggests that CLDN18.2 in CMA is unlikely to be a classic oncogenic driver; instead, it is better viewed as a therapeutically accessible, lineage-associated membrane antigen. This review summarizes the biological rationale, expression data, companion diagnostic issues, therapeutic platforms, and translational barriers relevant to CLDN18.2 in CMA. We propose a framework centered on lineage reprogramming, antigen enrichment, assay standardization, and therapeutic translation. Current evidence supports CLDN18.2 as a candidate biomarker for biomarker-enriched investigation, not as an established therapeutic target in CMA. Future work should prioritize CMA-specific preclinical models, standardized membranous immunohistochemistry (IHC) scoring, and prospective trials that integrate CLDN18.2 expression with molecular subtype and tumor microenvironmental features.

## 1 Introduction

Colorectal mucinous adenocarcinoma (CMA) is a distinct histologic subtype of colorectal cancer, generally defined by extracellular mucin accounting for at least 50% of tumor volume (Han et al., 2024). It accounts for approximately 10%–20% of all colorectal cancers (Wang X. et al., 2024). Compared with non-mucinous colorectal adenocarcinoma, CMA more often arises in the proximal colon, is diagnosed at a relatively advanced stage, and is associated with poor differentiation, signet-ring cell components, peritoneal dissemination, and less favorable clinical outcomes (Wang X. et al., 2024); (Yuan et al., 2025). Molecularly, CMA frequently overlaps with deficient mismatch repair/high microsatellite instability (dMMR/MSI-H), BRAF V600E mutation, CpG island methylator phenotype-high (CIMP-high) status, and serrated pathway-associated backgrounds, producing a clinicopathologic profile that differs from non-mucinous colorectal adenocarcinoma (Wang X. et al., 2024); (Wang JD. et al., 2024). Importantly, CMA is highly heterogeneous. Variation in mucin proportion, signet-ring cell infiltration, mixed histological components, and molecular background can translate into divergent tumor and clinical behavior. CMA therefore should not be treated as a homogeneous entity in target discovery or clinical analyses.

From a clinical translational perspective, CMA is more than a mucin-rich morphologic variant. Abundant extracellular mucin can remodel the tumor microenvironment, impede immune-cell infiltration and drug penetration, dilute tumor cellularity, and disrupt the spatial continuity of antigen expression. These features can cause conventional biopsy or tissue microarray-based testing to underestimate the true prevalence and distribution of membrane antigens, with direct implications for targeted agents, antibody-drug conjugates (ADCs), and engineered immune-cells. In addition, advanced CMA often progresses through mucinous peritoneal dissemination, for which conventional response evaluation criteria often fail to capture biologic behavior or treatment response. Accordingly, clinical research and precision oncology strategies for CMA should integrate three dimensions: histologic subtype, molecular context, and microenvironmental barriers (Keller and Averkiou, 2022); (Yuan et al., 2023); (Luo et al., 2019). Clinically, advanced CMA remains challenging to treat. Mucinous mCRC has shown lower response rates to first-line chemotherapy than non-mucinous disease (18.4% vs. 49.0%) (Catalano et al., 2009), while dMMR/MSI-H mucinous tumors also showed a lower response rate to immune checkpoint inhibitors (48.0% vs. 65.1%) (Nasca et al., 2026). In addition, synchronous mucinous peritoneal metastases were associated with shorter disease-specific survival after CRS plus HIPEC (22.4 vs. 36.3 months) (Herold et al., 2022). These findings highlight the persistent unmet need for more effective treatment strategies in advanced CMA.

The current precision oncology framework for advanced colorectal cancer includes several targetable alterations, including dMMR/MSI-H, BRAF V600E, HER2 amplification, KRAS G12C, and NTRK fusion. However, this framework has largely been developed in conventional colorectal adenocarcinoma and has not been tailored to the pathologic phenotype of CMA (Wang et al., 2025); (Benson et al., 2024). Actionable driver alterations in CMA are scattered and heterogeneous, and many CMA tumors are proficient mismatch repair/microsatellite stable (pMMR/MSS) and lack established targetable drivers. Existing treatment strategies therefore do not fully address clinically relevant features of CMA, including the mucin barrier and lineage-specific differentiation. Identifying membrane-localized, lineage-associated, and therapeutically accessible antigens is therefore an important step toward a more refined precision-treatment framework for CMA.

CLDN18.2 is the gastric isoform of the CLDN18 gene and is regulated by tissue-specific promoter activity. Under physiologic conditions, it is stably expressed in mature gastric epithelial cells, whereas normal colorectal mucosa shows low or absent expression (Cao et al., 2022). Its extracellular domains can become accessible on the tumor-cell membrane, supporting its potential as a therapeutic antigen (Huang et al., 2025). CLDN18.2-directed therapy has made substantial progress in gastric and GEJ adenocarcinomas, where phase 3 trials have shown that zolbetuximab improves survival outcomes in patients with CLDN18.2-positive advanced disease (Shah et al., 2023).

Recent large pathology-based cohort studies have shown that CLDN18.2 expression is low in unselected conventional colorectal adenocarcinoma but enriched in CMA, mucin-rich CRC, and colorectal tumors with gastric-type or foregut-like differentiation, particularly in subgroups defined by proximal colonic location, dMMR/MSI-H status, or BRAF V600E mutation (Yan et al., 2025); (McHugh et al., 2025). Previous CLDN18.2 reviews have largely focused on gastric/GEJ cancer or broader gastrointestinal therapeutic development, with colorectal cancer generally discussed within multi-tumor contexts. In contrast, this review focuses specifically on whether CLDN18.2 can serve as a clinically actionable biomarker in CMA, where mucin-rich architecture, low tumor-cell density, and spatial heterogeneity may affect both biomarker assessment and therapeutic accessibility. We therefore integrate lineage-associated biology, CMA/CRC pathology evidence, sampling and drug-delivery barriers, and platform-specific therapeutic considerations into a translational framework linking target expression to patient selection and clinical development. Table 1 summarizes key differences among CMA, conventional CRC, and gastric/GEJ adenocarcinoma in morphology, molecular features, and the rationale for CLDN18.2 targeting. Rather than extrapolating directly from gastric cancer, we focus on CMA-specific mechanisms of target expression, patient selection, diagnostic standards, and clinical feasibility.

**TABLE 1:** Clinical, pathologic, and molecular features relevant to CLDN18.2 targeting in CMA, conventional colorectal adenocarcinoma, and gastric/GEJ adenocarcinoma.

Feature	Colorectal mucinous adenocarcinoma (CMA)	Conventional colorectal adenocarcinoma	Gastric cancer
Typical location	More often proximal/right-sided	Often distal/left-sided or rectal, with cohort variability	Stomach or gastroesophageal junction
Histology	Extracellular mucin constitutes at least 50% of tumor volume	Non-mucinous gland-forming adenocarcinoma	Intestinal, diffuse, or mixed histologic patterns
Core molecular features	Enriched for dMMR/MSI−H, BRAF V600E, CIMP-high, and serrated pathway biology	pMMR tumors are more common; APC/KRAS pathway alterations are frequent	Clinically relevant subgroups include HER2−positive, EBV-positive, MSI/dMMR, and CLDN18.2−positive disease
CLDN18.2 expression	Enriched in selected mucinous, proximal, and dMMR-related subgroups	Low overall expression in unselected cases	Clinically relevant in selected CLDN18.2−positive populations
Therapeutic implications	Candidate enrichment biomarker; clinical validation is still required	Not suitable as a broad-spectrum target in unselected CRC	Clinically validated therapeutic target in selected patients

## 2 Biological basis for CLDN18.2 enrichment in CMA

### 2.1 CLDN18.2 as a lineage-associated membrane antigen for therapeutic technologies

Claudins are core proteins that maintain epithelial tight-junction structure and function. Through alternative promoter usage, CLDN18 encodes two tissue-specific isoforms: CLDN18.1 and CLDN18.2 (Nakayama et al., 2024). CLDN18.2 is a four-pass transmembrane protein, and its first extracellular loop is a key structural domain recognized by several therapeutic modalities, including monoclonal antibodies, antibody-drug conjugates (ADCs), chimeric antigen receptor T cells (CAR-T cells), and T-cell engagers (Nakayama et al., 2024); (Murtazaalieva et al., 2026); (Barrett et al., 2024); (Zhong et al., 2022).

The translational value of CLDN18.2 derives primarily from the therapeutic accessibility of its extracellular loop. Unlike nuclear or cytoplasmic markers, cell-surface membrane antigens can be directly recognized by monoclonal antibodies, ADCs, and engineered immune cells, making them attractive targets for drug delivery and immune-mediated therapy (Nakayama et al., 2024). However, the epitopes recognized by diagnostic antibodies and therapeutic constructs are not necessarily identical, and the corresponding reagents can differ in binding affinity, conformational dependence, and interpretation of membranous IHC staining. Therefore, CLDN18.2 assessment in CMA should not only determine whether the protein is present, but should also quantify its abundance and spatial distribution on the tumor-cell membrane.

In normal gastric mucosa, CLDN18.2 is largely confined to epithelial tight-junction regions, where circulating therapeutic agents have limited access. In malignant tissue, disruption of epithelial polarity and tight-junction integrity can expose CLDN18.2 membrane epitopes, creating a therapeutic window for targeted intervention (Carstens et al., 2025); (Fassan et al., 2024). This pattern—antigen sequestration in normal tissue but antigen exposure in tumor tissue—underpins the appeal of CLDN18.2 as a solid-tumor target. Nonetheless, this therapeutic window is not absolute. In particular, platforms with strong immune activation or highly cytotoxic payload release require careful evaluation of gastric mucosal on-target/off-tumor toxicity.

Available evidence suggests that CLDN18.2 expression in CMA is not driven by classic genomic events such as amplification, fusion, or structural rearrangement. A more plausible interpretation is that CLDN18.2 represents a membrane phenotype associated with gastric-type or foregut-like differentiation, rather than a driver event required for tumor proliferation (Yan et al., 2025); (McHugh et al., 2025). This distinction has direct translational implications. CLDN18.2-directed development in CMA should not simply follow the inhibitor paradigm used for oncogenic drivers; it should instead focus on antigen-screening strategies, quantitative standards for membranous expression, spatial heterogeneity, and drug-delivery performance.

### 2.2 Gastric-lineage reprogramming as a working model for CLDN18.2 enrichment

In this review, “gastric-lineage reprogramming” refers to the aberrant acquisition of gastric-type mucin markers, foregut-like differentiation programs, and gastric-derived membrane proteins such as CLDN18.2 during colorectal tumor evolution. It does not imply that CMA fully acquires the biological identity of gastric cancer. CMA is commonly associated with abnormal mucin differentiation and serrated pathway activation. Some tumors express gastric-type mucin markers such as MUC5AC and MUC6, suggesting partial activation of gastric-type or foregut-like lineage programs under specific molecular contexts (Wang JD. et al., 2024); (Yan et al., 2025); (Walsh et al., 2013). On this basis, aberrant CLDN18.2 expression in CMA may reflect remodeling of tumor-cell lineage state rather than simply increased mucin production, although this interpretation remains hypothetical.

The serrated neoplasia pathway provides a biologically plausible context for lineage remodeling in CMA (Wang X. et al., 2024); (Wang JD. et al., 2024). Core molecular features include BRAF or KRAS driver mutations, sustained MAPK pathway activation, CIMP-high epigenetic status, and methylation-mediated silencing of multiple tumor suppressor genes. MLH1 promoter methylation can further lead to a dMMR/MSI-H phenotype (Wang JD. et al., 2024). Histologically, tumors arising through this pathway are often right-sided and overlap with mucinous morphology, poor differentiation, and special differentiation phenotypes. This sequence—from molecular alteration and epigenetic dysregulation to lineage remodeling—provides a biologically plausible framework for interpreting aberrant CLDN18.2 expression in a subset of CMA, but the causal links among these events have not been established. However, direct functional evidence linking CIMP-high status, MLH1 methylation, gastric-type mucin markers, and CLDN18.2 expression remains insufficient. The enrichment of CLDN18.2 in BRAF V600E-mutant and dMMR CRC may therefore reflect a shared serrated/CIMP-high and gastric-lineage phenotype rather than direct regulation by either molecular alteration (McHugh et al., 2025); (Takamizawa et al., 2024).

Cross-tumor expression patterns further support an association between CLDN18.2 and gastric-type or foregut-like lineage states. Beyond gastric cancer, CLDN18.2 expression has been reported in pancreatic ductal adenocarcinoma (Lyu et al., 2024); (Arseneau et al., 2025), cholangiocarcinoma (Kinzler et al., 2025); (Shin et al., 2026), ovarian mucinous carcinoma (Wang et al., 2023); (Wagner et al., 2024), lung adenocarcinoma or mucinous lung adenocarcinoma (Liu et al., 2022), and some small bowel adenocarcinomas (Fujii et al., 2025). These tumor types exhibit gastric-type, foregut-like, or upper gastrointestinal differentiation features to varying degrees. By contrast, CLDN18.2 positivity is markedly uncommon in unselected conventional colorectal adenocarcinoma (Yan et al., 2025). Thus, CLDN18.2 in CMA is unlikely to be merely a pan-mucinous marker; rather, it can help define a mucinous tumor subgroup with gastric-type or foregut-like differentiation. The upstream regulatory mechanisms of CLDN18.2 in CMA remain incompletely defined. Current knowledge is largely extrapolated from gastric, pancreatic, and other gastrointestinal tumors. Lineage-specific transcriptional regulation, epigenetic remodeling, CIMP-high features, and serrated pathway activation have been proposed as potential contributors (Yan et al., 2025); (Lim et al., 2025); (Tao et al., 2023); (Chen et al., 2026). Among candidate regulators, the GATA and FOXA families are important transcription factors in gastrointestinal and foregut lineage differentiation, with GATA-mediated regulatory programs well characterized in gastric cancer (Begolli et al., 2024). However, direct data on the expression of these transcription factors in CMA remain sparse, and no consistent CMA-specific enrichment or direct regulation of CLDN18.2 has been established. Their involvement in gastric-lineage reprogramming of CMA should therefore be regarded as a hypothesis derived from cross-tumor lineage biology rather than a demonstrated CMA-specific mechanism.

Taken together, gastric-lineage reprogramming should currently be viewed as a working model for CLDN18.2 enrichment in CMA, not as a causal mechanism proven by functional experiments. CLDN18.2 positivity suggests partial remodeling of tumor lineage differentiation; it does not mean that the tumor has acquired a gastric cancer-like phenotype or that CLDN18.2 actively drives CMA progression. A more rigorous interpretation is that, under the combined influence of serrated pathway activation, CIMP-high epigenetic context, and abnormal mucin differentiation programs, a subset of CMA cells may acquire gastric-type or foregut-like features, with CLDN18.2 serving as a detectable membrane marker of this lineage state.

Functionally, it remains unknown whether CLDN18.2 contributes to CMA behaviors such as local invasion, peritoneal dissemination, or immune escape. Studies in other gastrointestinal tumors, including pancreatic and cholangiocarcinoma, suggest potential roles for CLDN18.2 in cell adhesion, migration and invasion, as well as modulation of the tumor immune microenvironment (Zhou et al., 2025); (K et al., 2025). However, these findings require validation in CMA-specific models.

If CLDN18.2 is primarily a lineage-dependent antigen rather than a tumor driver, therapeutic success will depend more on membrane antigen density, spatial coverage, and delivery efficiency than on tumor-cell dependence on CLDN18.2 signaling. Based on these evidence boundaries, Figure 1 presents a hypothetical biological model in which mucinous morphology, serrated pathway biology, and CIMP-high-related backgrounds may be associated with a partial gastric-type or foregut-like lineage state, with CLDN18.2 representing a detectable membrane phenotype.

**FIGURE 1:**
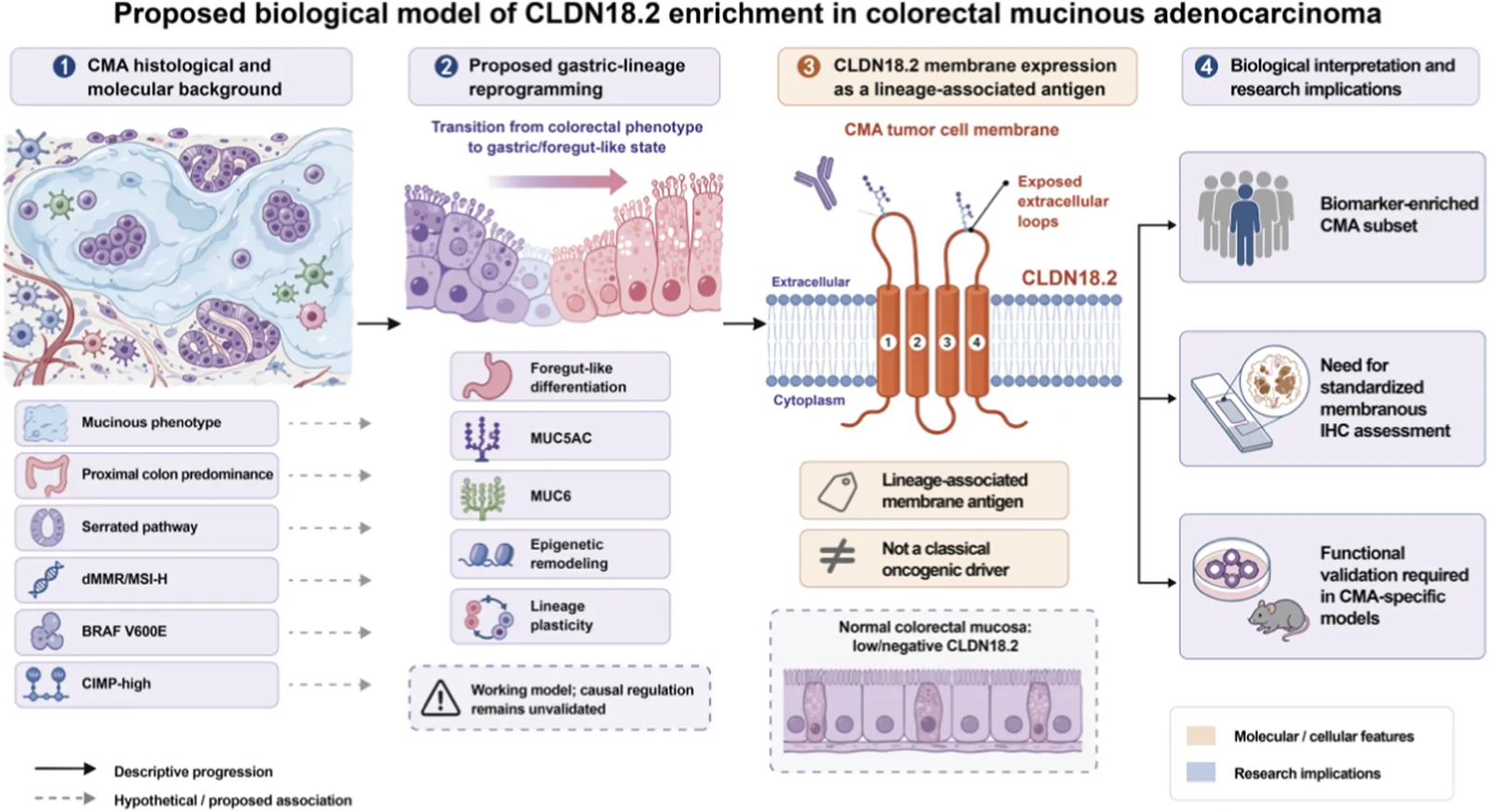
Proposed biological model of CLDN18.2 enrichment in colorectal mucinous adenocarcinoma. Colorectal mucinous adenocarcinoma (CMA) is characterized by abundant extracellular mucin, low-density tumor-cell clusters, and frequent overlap with serrated pathway biology, dMMR/MSI-H, BRAF V600E mutation, and CIMP-high features. These contexts are consistent with partial gastric-lineage or foregut-like reprogramming, reflected by markers such as MUC5AC and MUC6. In this model, Tumor-related loss of polarity can expose CLDN18.2 extracellular loops, allowing membranous IHC detection and potential therapeutic access. The causal links among serrated pathway activation, epigenetic remodeling, lineage reprogramming, and CLDN18.2 expression require validation in CMA-specific models.

## 3 Evidence for CLDN18.2 expression and companion diagnostic challenges in CMA

### 3.1 Low expression in unselected CRC and enrichment in mucinous subtypes

Direct pathology-based evidence from CRC and CMA cohorts indicates that CLDN18.2 is expressed at low frequency in unselected conventional colorectal adenocarcinoma but is enriched in CMA and mucin-rich CRC. Yan et al. analyzed 423 colorectal adenocarcinoma samples by immunohistochemistry and reported an overall CLDN18.2 positivity rate of only 2.4% in conventional colorectal adenocarcinoma, compared with 27.3% in the mucinous adenocarcinoma subgroup (Yan et al., 2025). Another large cohort of 1,404 digestive tract adenocarcinomas, including 1,175 colorectal cancers, used tissue microarray-based immunohistochemistry with the Ventana CLDN18 (43-14A) RxDx assay and reported an overall CLDN18.2 positivity rate of 3.4% in CRC, with positive cases concentrated in tumors with mucinous subtype, proximal colonic primary site, and dMMR-related phenotype (McHugh et al., 2025). Together, these studies indicate that CLDN18.2 is not a broadly expressed CRC target but is enriched in molecularly and histologically defined subgroups.

This differential expression pattern has important implications for translational research. In CLDN18.2-directed studies of CRC, careful population selection is likely more informative than reporting overall positivity in an unselected cohort. Broad testing across pan-CRC populations can yield low positivity rates and obscure the potential value of CLDN18.2 in biologically defined subtypes. By contrast, focusing on proximal colonic lesions, dMMR/MSI-H CMA, or mucinous tumors with BRAF V600E mutation is likely to more efficiently identify tumors with CLDN18.2-positive membranous expression and potential therapeutic relevance. Development of CLDN18.2 as a CRC target should therefore move from broad, unselected screening toward enrichment-based strategies guided by pathologic subtype and molecular features.

Nevertheless, positivity rates across existing studies should not be compared directly without caution. Studies differ in diagnostic antibody clones, IHC platforms, thresholds for membranous staining, criteria for positive-cell proportion, and sampling methods, including tissue microarray versus whole-section staining. These methodological differences are particularly consequential in CMA, where abundant mucin pools and sparse tumor-cell distribution can amplify sampling bias. Tissue microarrays or single-site biopsies can fail to capture the full spatial distribution of CLDN18.2 expression and can underestimate target coverage across the tumor. At present, CLDN18.2 enrichment in CMA should be interpreted as a reproducible biological trend rather than as a quantitatively settled epidemiologic estimate. Notably, McHugh et al. (McHugh et al., 2025) applied the clinically validated Ventana CLDN18 (43-14A) RxDx assay and the SPOTLIGHT/GLOW criterion of ≥75% moderate-to-strong membranous staining to 1,175 CRCs, showing that CLDN18.2-positive tumors were significantly enriched for mucinous features. Similarly, a large CRC cohort evaluated with clone 43-14A found CLDN18.2 expression to be associated with mucinous histology. However, CMA-specific analytical validation of the 43-14A assay remains limited (Takamizawa et al., 2024).

Based on current evidence, CLDN18.2 is not suitable for broad screening across all colorectal cancers. Its more precise role is as a candidate stratification biomarker for selected biological subtypes, particularly CMA. Importantly, current clinical evidence is insufficient to support CLDN18.2 as a prognostic biomarker in CMA. Data are lacking to establish clear associations between CLDN18.2 expression and recurrence, peritoneal dissemination, progression-free survival, or overall survival. Table 2 summarizes major pathology studies and the interpretive boundaries of CLDN18.2 expression in CRC, CMA, and related gastrointestinal tumors.

**TABLE 2:** Representative studies of CLDN18.2 expression in CMA/CRC and related gastrointestinal adenocarcinomas.

Representative study	Tumor type / cohort	Cohort size	Antibody / clone	IHC platform / specimen	Positivity cutoff	CLDN18.2 expression frequency
Yan et al. (2025)	CRC cohort with an additional CMA series	CRC TMA: n=423; CMA: n=22	Mouse monoclonal LBP−CLDN18.2	Platform not reported; CRC tissue microarray (TMA) and separate CMA tissue specimens	Any membranous staining was considered expression; high expression was defined as ≥2+ staining in ≥50% of tumor cells	Any expression: CRC, 10/423 (2.4%); CMA, 6/22 (27.3%). High expression: CRC, 2/423 (0.47%); CMA, 3/22 (13.64%)
McHugh et al. (2025)	Digestive tract adenocarcinomas with a large CRC cohort	Total: n=1,404; CRC: n=1,175 (test cohort, n=576; validation cohort, n=599)	43–14A	Ventana CLDN18 (43–14A) RxDx assay; TMA, 2–3 cores per tumor	Moderate-to-strong membranous staining in ≥75% of tumor cells	CRC: 40/1,175 (3.4%)
Fujii et al. (2025)	Small bowel adenocarcinoma	n=51	43–14A, Roche Diagnostics	Platform not reported; FFPE surgical resection or biopsy specimens	Moderate-to-strong membranous staining in ≥75% of tumor cells	SBA, 18/51 (35%)
Takamizawa et al. (2024)	Stage I−III surgically resected CRC	n=805	43–14A, Roche Ventana	HISTO STAINER automated immunostainer (Nichirei Biosciences); TMA constructed from surgical specimens	Moderate-to-strong membranous staining in ≥1% of tumor cells	CRC, 17/805 (2.1%)
Hashimoto et al. (2025)	Pan-solid-tumor MONSTAR−SCREEN-2 cohort	IHC: n=349 (CRC, n=37); WTS: n=2,191	ab222512 (EPR19202), Abcam	Platform not reported; 4−μm FFPE sections	≥75%, ≥40%, and ≥25% tumor-cell thresholds evaluated; ≥40% used as the principal threshold for IHC−WTS correlation	CRC: ≥75%, 2.7%; ≥40%, 8.1%; ≥25%, 8.1%

Reported positivity frequencies and definitions are presented according to the criteria used in the original studies. Studies involving CRC, or CMA, provide direct disease-relevant evidence, whereas studies of other gastrointestinal tumors are included only as extrapolative context. Direct numerical comparisons should be interpreted cautiously because of differences in antibody reagents, IHC, platforms, specimen types, sampling strategies, tumor populations, and positivity thresholds.

### 3.2 Establishing CMA-specific companion diagnostic standards for CLDN18.2

Immunohistochemistry (IHC) remains the most practical approach for clinical screening of CLDN18.2 expression. In gastric cancer, relatively mature systems for evaluating membranous CLDN18.2 staining have been established; however, these gastric cancer-based scoring standards have not been validated in CMA, and their direct application to CMA has important limitations (Nakayama et al., 2024); (Fassan et al., 2024). CMA is characterized by abundant extracellular mucin, low tumor-cell density, and pronounced spatial heterogeneity. CLDN18.2 expression can differ among tumor cells floating within mucin pools, cells at the invasive front, gland-forming regions, and poorly differentiated components. Under these conditions, tissue microarrays, single-site biopsies, or limited regional sampling can fail to capture the overall abundance and spatial continuity of membranous expression, leading to biased classification of target status.

Spatial heterogeneity of CLDN18.2 should be considered at both intratumoral and interlesional levels. Within an individual tumor, regional variation in staining intensity and the proportion of positive tumor cells may cause different areas of the same lesion to cross or fall below a predefined positivity threshold. Across disease sites, CLDN18.2 expression may also differ between the primary tumor and metastatic lesions, introducing an additional source of biomarker misclassification. In metastatic gastric cancer, intratumoral heterogeneity was observed in 38.5% of surgical specimens, while 25.2% of paired primary and metastatic lesions were discordant for CLDN18.2 positivity (Choi et al., 2024). A separate paired gastric/GEJ cohort similarly reported intratumoral heterogeneity in 25.5% of primary tumors and 19.6% of metastatic lesions, with approximately 20% discordance between paired primary and metastatic samples (Son et al., 2025). Importantly, heterogeneity may also be site dependent: peritoneal metastases showed higher CLDN18.2 positivity and positive concordance with primary tumors than liver metastases in one metastatic gastric cancer cohort (Choi et al., 2024). Although these data are derived from gastric/GEJ cancer and cannot be directly extrapolated to CMA, they demonstrate that CLDN18.2 expression should not be assumed to be spatially stable within a tumor or across metastatic sites.

Interpretation of CLDN18.2 testing in CMA should therefore prioritize specific membranous staining rather than isolated nonspecific cytoplasmic signal and should account for spatial heterogeneity both within and between tumor lesions. For clinical trial design, recently obtained tumor samples that best represent the therapeutic target lesion should be preferred. Because regional variation in CLDN18.2 expression can introduce sampling bias, whole-section staining or multi-region assessment should be used whenever feasible; when both primary and metastatic tissues are available, their concordance should also be evaluated (Fassan et al., 2024); (Wagner et al., 2024). If only archival primary-tumor tissue is available, its ability to represent CLDN18.2 expression across metastatic disease should not be assumed, particularly in CMA with peritoneal dissemination. Future CMA studies should therefore prospectively assess intratumoral spatial heterogeneity and paired primary-metastatic concordance using consistent antibody clones, staining platforms, and positivity criteria.

Different CLDN18.2-targeted platforms can require different levels and patterns of antigen expression. Accordingly, a CMA-specific companion diagnostic framework should include three sequential components: analytical standardization, spatially representative assessment, and platform-specific clinical calibration. First, analytical standardization should harmonize diagnostic antibody clones and IHC platforms and apply interpretation criteria centered on specific membranous staining, with standardized reporting of staining intensity and positive-cell proportion. Second, spatially representative assessment should incorporate whole-section or multi-region evaluation when feasible, document specimen source and lesion type, and account for intratumoral heterogeneity and primary-metastatic concordance. Third, in the absence of CMA-specific efficacy data, a single universal positivity threshold would be premature. Candidate thresholds should instead be calibrated for individual therapeutic platforms by prospectively correlating quantitative and spatial IHC variables with treatment response and clinical outcomes. CLDN18.2 assessment in CMA should therefore move beyond a binary “positive” or “negative” result toward a quantitative, spatially resolved, and platform-calibrated evaluation system.

To support this framework, future CLDN18.2 IHC studies should report antibody clone, staining platform, membranous staining intensity, positive-cell proportion, sampling region, specimen type (whole section or tissue microarray), and lesion type (primary or metastatic). The relationship between sampled tumor regions and mucin pools, stromal areas, and the invasive front should also be documented, while MMR status, BRAF V600E status, mucin proportion, and primary tumor location may be integrated into exploratory composite biomarker models. Because CLDN18.2 expression varies across digestive tract tumor subtypes and histologic backgrounds, future studies should report subtype-specific results stratified by platform, threshold, and sample type rather than overall positivity alone (Hashimoto et al., 2025).

In summary, the main bottleneck in CLDN18.2 companion diagnostics for CMA is not whether the target can be detected, but whether the result is reliable, reproducible, and clinically predictive. Only an analytically standardized, spatially representative, and platform-calibrated IHC framework validated against prospective efficacy data can move CLDN18.2 from a subtype-enriched expression pattern toward a clinically actionable biomarker for patient stratification and targeted therapy.

## 4 Translational potential and limitations of CLDN18.2-targeted therapy in CMA

### 4.1 Evidence from gastric cancer cannot be considered direct clinical evidence in CMA

Gastric and gastroesophageal junction (GEJ) adenocarcinomas currently provide the strongest clinical evidence for CLDN18.2-targeted therapy. The pivotal phase 3 GLOW and SPOTLIGHT trials, which enrolled 507 and 565 patients, respectively, evaluated first-line zolbetuximab combined with CAPOX or mFOLFOX6 in patients with CLDN18.2-positive, HER2-negative advanced gastric or GEJ adenocarcinoma and showed significant improvements in progression-free survival and overall survival (Shah et al., 2023); (Shitara et al., 2023). These findings provide several lessons for CLDN18.2-directed development. First, CLDN18.2 is a druggable solid-tumor membrane target that can support IHC-based patient selection. Second, CLDN18.2 targeting can generate quantifiable survival benefit when combined with standard chemotherapy in an appropriate tumor context. Third, CLDN18.2 should be incorporated into multidimensional treatment stratification alongside established biomarkers such as HER2, PD-L1, and MSI rather than used as an isolated therapeutic determinant (Kono et al., 2024).

However, the success of CLDN18.2-targeted therapy in gastric cancer should not be directly extrapolated to CMA. CMA and gastric cancer differ in tissue origin, lineage programs, mucinous architecture, metastatic pattern, tumor microenvironment, and standard systemic therapy. The most defensible conclusion at present is that CLDN18.2 is expressed in a subset of CMA and could serve as a membrane antigen for targeted intervention, but no clinical evidence yet demonstrates reproducible antitumor efficacy of CLDN18.2-directed therapy in CMA. Maintaining this evidence boundary is essential to avoid overinterpretation and to preserve translational rigor.

### 4.2 CLDN18.2-targeted therapeutic platforms: opportunities and constraints

Multiple CLDN18.2-targeted platforms are under development, including monoclonal antibodies, ADCs, CAR-T cells, T-cell engagers, T-cell antigen-coupled technologies, mRNA-encoded antibodies, and tumor vaccines (Nakayama et al., 2024); (Dominguez et al., 2025); (Chen et al., 2023). However, these approaches differ substantially in clinical maturity: zolbetuximab and satricabtagene autoleucel have achieved regulatory approval in gastric/GEJ adenocarcinoma, selected CLDN18.2-targeted ADCs have entered late-stage clinical development, TAC-based cellular therapy is in early clinical development, whereas DR30318 and vaccine-based strategies remain preclinical. These platforms share a dependence on CLDN18.2 accessibility at the tumor-cell membrane but differ in antigen-density requirements, tissue penetration, immune-activation intensity, toxicity profile, and tolerance of antigen heterogeneity. Unless otherwise specified, the therapeutic and mechanistic evidence discussed below is derived from gastric/GEJ cancer or other solid-tumor models and should therefore be considered extrapolative rather than direct evidence of efficacy in CMA. Because no CLDN18.2-targeted platform has yet been validated in CMA, their translational relevance should be evaluated against CMA-specific constraints, including abundant extracellular mucin, low tumor-cell density, discontinuous antigen distribution, stromal barriers, and peritoneal dissemination.

Among available platforms, monoclonal antibodies such as zolbetuximab have the most mature clinical evidence. Their antitumor activity is mediated primarily through antibody-dependent cellular cytotoxicity and complement-dependent cytotoxicity (Shah et al., 2023). Although this strategy has been validated in gastric cancer, macromolecular antibodies can be limited by vascular extravasation, diffusion distance, interstitial pressure, and extracellular matrix barriers (Keller and Averkiou, 2022); (Yuan et al., 2023). These barriers are particularly relevant in mucin-rich CMA. Monoclonal antibody-based targeting is most biologically plausible when CLDN18.2 membranous expression is strong, spatially continuous, and relatively homogeneous. If expression is patchy or tumor cells are separated by abundant mucin, drug-target contact can be reduced, potentially weakening antitumor activity.

CLDN18.2-targeted ADCs span early-to late-stage clinical development, with tecotabart vedotin (LM-302) having entered a phase III trial in gastric/GEJ adenocarcinoma (NCT06351020). ADCs can deliver cytotoxic payloads to CLDN18.2-positive tumor cells, and payload bystander effects can partially offset antigen heterogeneity (Bai et al., 2025); (Tsuchikama et al., 2024). This feature is conceptually attractive for CMA, where tumor-cell density is often low and target expression discontinuous. Even if only a subset of tumor cells expresses CLDN18.2, locally released payloads can affect nearby CLDN18.2-negative cells. Nevertheless, ADC efficacy in CMA may be constrained at two sequential transport steps: penetration of the intact ADC to CLDN18.2-positive tumor cells and subsequent diffusion of the released payload through mucin-rich extracellular spaces. Recent intratumoral imaging has shown that the binding-site barrier can restrict ADC penetration and that bystander activity only partially overcomes this spatial limitation (Wei et al., 2024). In a colorectal cancer-oriented mucus model containing MUC2 and MUC5AC, mucus composition and concentration altered pore size and nanoparticle diffusivity, supporting the possibility that mucin-rich architecture can further constrain intratumoral transport (Zhao et al., 2024). Whether released low-molecular-weight ADC payloads can effectively traverse MUC2-rich mucin pools in CMA remains unknown. Mucin-degrading approaches may therefore warrant investigation as an adjunct to improve intratumoral drug distribution, although no study has yet demonstrated that mucinase treatment enhances the efficacy of CLDN18.2-targeted ADCs in CMA (Geng et al., 2026).

Cellular and T-cell–redirecting approaches differ in clinical maturity. Satricabtagene autoleucel (satri-cel; CT041) has been approved in China for patients with CLDN18.2-positive, HER2-negative advanced gastric/GEJ adenocarcinoma after failure of at least two prior lines of therapy. TAC01-CLDN18.2 is being evaluated in a phase I/II trial (NCT05862324), whereas DR30318 remains preclinical (Qi et al., 2024). CLDN18.2-directed CAR-T cells, T-cell engagers, and T-cell antigen-coupled engineered T cells can redirect immune effector cells to recognize and eliminate CLDN18.2-positive tumor cells (Shah et al., 2023); (Ma et al., 2024); (Xu et al., 2025). However, mucin and stromal components in CMA can restrict engineered T-cell infiltration into tumor parenchyma. Engineering immune effector cells with glycocalyx- or matrix-remodeling activity represents a potential strategy to overcome this physical barrier. Park et al. demonstrated that engineering effector cells to display glycocalyx-editing enzymes, including mucinases and sialidases, enhanced cytotoxic activity against mucin-bearing tumor cells (Park et al., 2024). Complementarily, heparanase-engineered CAR-T cells recently showed markedly improved extracellular-matrix penetration and antitumor activity in 2D and 3D colorectal cancer models (Zhu et al., 2026). Mucinase-based remodeling has also increased CD8^+^ T-cell infiltration in murine colorectal cancer models (Geng et al., 2026). These findings provide a rationale for exploring locally controlled mucin-degrading functions in CLDN18.2-directed cellular therapies for MUC2-rich CMA, although direct evidence for MUC2-specific enzyme-engineered CLDN18.2 CAR-T cells is currently lacking. Local immunosuppression, T-cell exhaustion, antigen-negative clonal escape, and gastric mucosal on-target/off-tumor toxicity are also important barriers. Before clinical testing in CMA, organoid, patient-derived xenograft, and peritoneal dissemination models that preserve mucinous and stromal features should be used to assess tissue penetration, tumor killing, and safety.

mRNA-encoded antibodies and CLDN18.2-targeted dendritic cell vaccines remain preclinical or early exploratory approaches (Bahr-Mahmud et al., 2023); (Zheng et al., 2026). mRNA-encoded antibodies could enable *in vivo* synthesis of functional antibodies, prolong exposure, and reduce limitations associated with repeated exogenous antibody administration (Bahr-Mahmud et al., 2023). CLDN18.2-targeted vaccine development also supports the concept that this antigen could be exploited for tumor immunotherapy (Zheng et al., 2026). However, neither platform has direct evidence in CMA. Future studies should determine whether these strategies can support efficient antigen presentation, initiate host antitumor immunity, and generate durable immune responses within a mucinous tumor microenvironment. Table 3 compares the mechanisms, potential relevance, and major limitations of CLDN18.2-targeted platforms in CMA.

**TABLE 3:** Development status and CMA-specific translational considerations of representative CLDN18.2-targeted therapeutic platforms.

Platform	Representative drug or approach	Development stage	Mechanism	Potential CMA-specific rationale	CMA-specific limitations
Monoclonal antibody	Zolbetuximab	Approved for gastric/GEJ adenocarcinoma; not validated in CMA	CLDN18.2−binding IgG1 antibody mediating ADCC and CDC	Provides a rationale for antibody-based targeting in CMA with high and spatially continuous membranous CLDN18.2 expression	No CMA-specific efficacy data; mucin and stromal barriers may restrict antibody penetration; on-target/off-tumor gastrointestinal toxicity remains relevant
ADC	Tecotabart vedotin (LM-302)	Late-stage clinical development (phase III); not validated in CMA	CLDN18.2 binding followed by internalization and cytotoxic payload release; potential bystander killing	Bystander killing may partially compensate for heterogeneous or patchy CLDN18.2 expression in CMA	No CMA-specific efficacy data; penetration of the intact ADC and diffusion of released payload through mucin-rich extracellular spaces remain uncertain
CAR−T	Satricabtagene autoleucel (satri-cel; CT041)	Approved in China for gastric/GEJ adenocarcinoma; not validated in CMA	Autologous CAR−T cells recognize CLDN18.2 and mediate direct cellular cytotoxicity	Direct cellular killing may be relevant to biomarker-selected, CLDN18.2−high CMA	No CMA-specific clinical evidence; mucin/stromal barriers, immunosuppression, antigen heterogeneity or escape, and gastric on-target/off-tumor gastrointestinal toxicity remain concerns
TAC	TAC01−CLDN18.2	Early clinical development (phase I/II); peer-reviewed evidence remains predominantly preclinical	CLDN18.2−targeted TAC co-opts the endogenous T-cell receptor complex to activate T cells	Preclinical activity at relatively low antigen levels may be relevant to CMA with lower-density or heterogeneous CLDN18.2 expression	No CMA-specific evidence; clinical efficacy remains unestablished and cellular trafficking through mucin/stroma may remain limiting
T-cell engager	DR30318	Preclinical	Tri-specific CLDN18.2×CD3×HSA engager mediating T-cell-dependent cytotoxicity	Provides an off-the-shelf T-cell–redirecting strategy that could be explored in CLDN18.2−positive CMA	No clinical or CMA-specific evidence; cytokine-mediated toxicity and effective access through mucin/stroma require evaluation
mRNA-encoded antibody	BNT141	Phase I/IIa trial terminated; development discontinued	mRNA−LNP drives *in vivo* production of an anti−CLDN18.2 IgG1 antibody	Provides an alternative strategy for systemic anti−CLDN18.2 antibody exposure	Clinical development was terminated; no CMA-specific evidence, and *in vivo* antibody production does not inherently overcome mucin/stromal penetration barriers
Vaccine	CLDN18.2−targeted dendritic cell vaccine	Preclinical	Antigen presentation and CLDN18.2−specific immune priming	Could generate systemic CLDN18.2−specific antitumor immunity in biomarker-selected CMA	Evidence remains preclinical and largely non−CMA; antigen presentation, T-cell trafficking, and immunosuppression in mucin-rich CMA remain unresolved

### 4.3 Histologic architecture of CMA and feasibility of CLDN18.2-targeted therapy

The histologic architecture of CMA directly affects the suitability of CLDN18.2-targeted platforms. Abundant extracellular mucin and stromal components can impede diffusion and infiltration of macromolecular antibodies, ADCs, and immune cells. Low tumor-cell density and discontinuous antigen expression can limit sustained antitumor effects from single-target therapies. In addition, peritoneal dissemination and mucinous peritoneal disease can reduce the likelihood that conventional intravenous administration achieves adequate drug exposure at target lesions. Thus, the central question is not only whether CLDN18.2 is expressed, but whether the antigen is present in drug-accessible tumor regions with adequate intensity, positive-cell proportion, and spatial coverage.

Therapeutic platform selection should therefore be matched to tumor architecture and metastatic pattern. Tumors with high-intensity membranous CLDN18.2 expression, a high positive-cell proportion, and continuous spatial distribution are likely more suitable for monoclonal antibodies or T-cell redirection. Tumors with patchy expression and marked heterogeneity are likely better suited to ADCs with bystander effects. For disease dominated by peritoneal dissemination, regional administration, enhanced local exposure, or combinations with stromal modulation deserve investigation. In short, CLDN18.2-targeted development in CMA should move from a binary “antigen-positive” approach to a multidimensional matching model that integrates antigen pattern, histologic architecture, metastatic site, and therapeutic platform.

Safety must also be considered. Physiologic CLDN18.2 expression in normal gastric mucosa creates an inherent risk of on-target/off-tumor toxicity, most commonly gastrointestinal events such as nausea, vomiting, and gastritis (Carstens et al., 2025); (Yamamoto et al., 2025). Platforms with stronger immune activation or highly cytotoxic payloads can carry additional risks of gastric mucosal injury, local inflammation, and systemic toxicity. Assessment of the therapeutic window in CMA should therefore consider tumor antigen density, spatial coverage, and normal-tissue toxicity thresholds.

### 4.4 Biomarker-enriched trials are needed to move CLDN18.2 from candidate target to clinical validation

The key task in CLDN18.2-directed development for CMA is not to reproduce the gastric cancer paradigm, but to build a disease-specific, testable translational pathway. Early-phase studies should use biomarker-enriched designs, prioritizing patients with proximal CMA, dMMR/MSI-H CMA, mucinous tumors harboring BRAF V600E mutation, high-risk peritoneal dissemination, or resistance to standard therapy. Baseline characterization should include CLDN18.2 membranous staining intensity, positive-cell proportion, spatial distribution, stromal architecture, MMR/MSI status, and BRAF mutation status.

Whether dMMR/MSI-H or BRAF V600E modifies sensitivity to CLDN18.2-directed therapy remains unknown. Their relevance may instead lie in defining the therapeutic context: dMMR/MSI-H supports consideration of immune checkpoint inhibition, whereas BRAF V600E may serve as an additional stratification variable rather than a presumed marker of CLDN18.2 sensitivity (Nasca et al., 2026); (McHugh et al., 2025); (Miyajima and Kawakami, 2025). ADC-mediated immunogenic cell death, immune activation by T-cell engagers, and CAR-T-driven microenvironmental remodeling also support combination or sequencing hypotheses. However, these approaches lack validation in CMA-specific models and clinical studies and should not yet be presented as standard treatment strategies.

In summary, CLDN18.2 research in CMA should move beyond expression description toward mechanistic validation, companion diagnostic development, and biomarker-enriched early-phase trials. Only after targeted-agent delivery, manageable safety, and reproducible antitumor activity are demonstrated in CMA can CLDN18.2 be advanced from a subtype-enriched marker to a clinically useful target for patient stratification and precision therapy. Figure 2 presents a simplified clinical-translational pathway for CLDN18.2-directed development in CMA. In this framework, candidate CMA populations with potential CLDN18.2 enrichment, such as proximal CMA, dMMR/MSI-H tumors, BRAF V600E-mutant tumors, mucin-rich tumors, or cases with peritoneal dissemination risk, undergo standardized membranous IHC assessment. Quantitative and spatial interpretation of CLDN18.2 expression may then support platform-specific matching, including monoclonal antibodies, ADCs, CAR-T/TAC-based cellular therapy, and T-cell engagers, followed by biomarker-enriched clinical validation.

**FIGURE 2:**
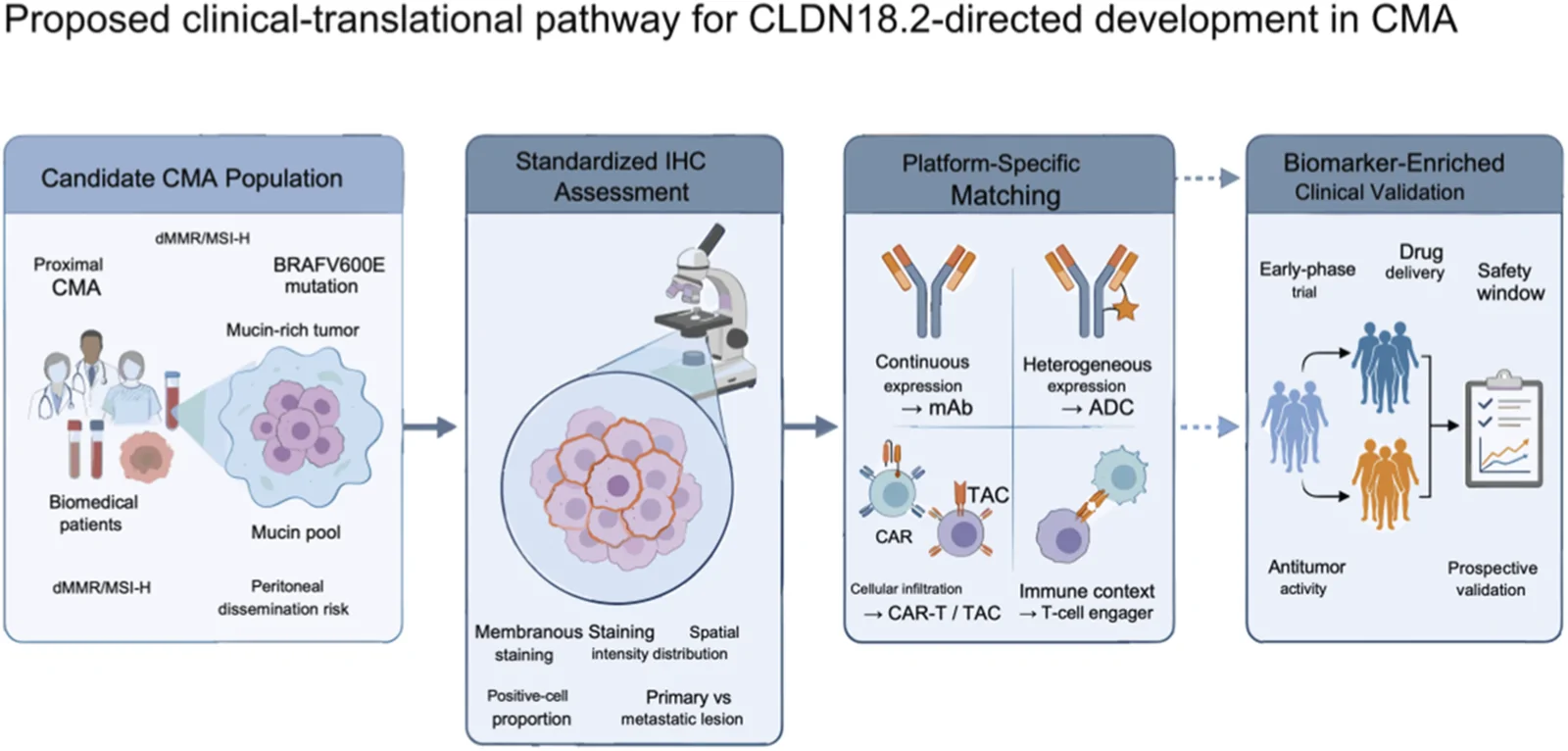
Proposed clinical-translational pathway for CLDN18.2-directed development in colorectal mucinous adenocarcinoma. Candidate CMA populations with potential CLDN18.2 enrichment should undergo standardized membranous IHC assessment, including staining intensity, positive tumor-cell proportion, spatial distribution, and primary-versus-metastatic lesion evaluation. These diagnostic variables may support platform-specific therapeutic matching, including monoclonal antibodies, antibody-drug conjugates, CAR-T/TAC-based cellular therapy, and T-cell engagers. Subsequent biomarker-enriched clinical validation should assess drug delivery, safety window, antitumor activity, prospective validation, and clinical benefit. The clinical utility of CLDN18.2-directed therapy in CMA remains unproven and requires further validation.

## 5 Current limitations and unresolved questions

Although pathology-based studies consistently suggest CLDN18.2 enrichment in CMA and mucin-rich CRC, the evidence base remains early. Most data come from retrospective pathology cohorts, tissue microarray-based analyses, and cross-tumor expression surveys. These studies establish an enriched expression pattern in defined histologic and molecular subgroups, but they do not show that CLDN18.2 predicts treatment response, recurrence risk, peritoneal dissemination, progression-free survival, or overall survival in patients with CMA.

Several biological questions remain unresolved. CLDN18.2 appears more consistent with a lineage-associated membrane antigen than with a classic oncogenic driver; however, the regulatory path from serrated pathway biology, CIMP-high status, and gastric-lineage reprogramming to CLDN18.2 expression has not been proven. It also remains unclear whether CLDN18.2 contributes to mucin production, tumor-cell adhesion, invasion, peritoneal spread, or immune escape, or whether it simply marks a particular lineage state. These unresolved questions require direct functional validation in CMA-specific experimental systems.

No CMA-specific CLDN18.2 scoring system has been established. Existing studies vary in antibody clone, IHC platform, membranous staining threshold, positive-cell cutoff, and sampling approach, which limits cross-study comparability. In CMA, mucin pools, low cellularity, and spatial heterogeneity make this problem especially important because tissue microarrays or single-site biopsies can misclassify target status.

Clinically, CLDN18.2-directed therapy has not been validated in CMA. Zolbetuximab has established CLDN18.2 as a druggable target in gastric/GEJ cancer, but CMA differs in lineage context, mucin-rich architecture, stromal barriers, metastatic pattern, and systemic treatment backbone. These differences can affect the delivery and activity of monoclonal antibodies, ADCs, CAR-T cells, and T-cell engagers. Evaluation in CMA should therefore integrate antigen density, spatial coverage, drug delivery, histologic architecture, immune context, and safety, rather than IHC positivity alone.

The optimal trial population is also undefined. It remains uncertain whether CLDN18.2 expression alone is sufficient for patient selection or whether histologic, molecular, and metastatic features should be incorporated into a composite enrichment strategy.

## 6 Discussion: from expression discovery to precision translation

Overall, the field should shift from documenting CLDN18.2 expression to testing clinical utility. The key question is whether the target is present in a therapeutically accessible pattern and in a patient population likely to benefit. Four steps are most important: CMA-relevant models, standardized diagnostics, enrichment-based trials, and platform-specific matching.

First, preclinical models should better recapitulate the histologic and microenvironmental features of CMA. Most pharmacologic and mechanistic data for CLDN18.2-targeted therapy come from gastric cancer, pancreatic cancer, and other solid-tumor systems (Barrett et al., 2024); (Xing et al., 2026); (Liu et al., 2023), which do not adequately model abundant extracellular mucin, low tumor-cell density, spatial antigen heterogeneity, peritoneal dissemination, or stromal barriers. Patient-derived organoids, patient-derived xenografts, three-dimensional co-culture systems, and peritoneal dissemination models should be prioritized (He et al., 2023); (Yau and Adriani, 2023), to assess antigen heterogeneity, drug penetration, local cytotoxicity, immune-cell access, and safety windows.

Second, CLDN18.2 testing in CMA should be standardized around therapeutically relevant variables. Rather than classifying tumors only as positive or negative, future assays should capture membranous staining intensity, positive-cell proportion, spatial distribution, specimen source, and lesion type. Because sparse tumor cells and discontinuous antigen expression can make limited sampling unreliable, digital pathology and multi-region sampling can help reduce reader variability and sampling bias. Clinically meaningful cutoffs should be defined prospectively by linking diagnostic variables to treatment outcomes.

Third, clinical enrollment should use biomarker enrichment rather than single-marker positivity alone. Early-phase trials should prioritize proximal CMA, dMMR/MSI-H CMA, mucinous tumors with BRAF V600E mutation, high-risk peritoneal dissemination, or disease resistant to standard therapy. CLDN18.2 should be treated as one part of a composite biological profile, incorporating MMR/MSI status, BRAF mutation phenotype, primary tumor location, mucin proportion, metastatic pattern, stromal architecture, and immune contexture.

Fourth, therapeutic platform selection should be matched to antigen pattern and tumor architecture. Tumors with high-intensity, spatially continuous membranous expression are likely better suited to monoclonal antibodies or T-cell redirection (Barrett et al., 2024); (Ma et al., 2024), whereas patchy or heterogeneous expression can favor ADCs with bystander killing (Tsuchikama et al., 2024). For disease dominated by peritoneal dissemination or mucinous peritoneal involvement, conventional intravenous delivery can be insufficient; regional delivery, enhanced local exposure, or stromal modulation deserves investigation.

Combination therapy is biologically attractive but remains speculative. The overlap between CLDN18.2-positive CMA, dMMR/MSI-H status, BRAF V600E mutation, and proximal colonic origin supports the hypothesis of combining or sequencing CLDN18.2-directed therapy with immune checkpoint inhibitors. ADC-mediated immunogenic cell death, T-cell engager- or CAR-T-mediated immune activation, and microenvironmental remodeling provide additional rationale (Barrett et al., 2024); (Tsuchikama et al., 2024). However, these concepts remain extrapolative; CMA-specific preclinical validation and clinical safety data are required.

In practical terms, the translational question is no longer simply whether CLDN18.2 is enriched in CMA, but whether its expression identifies a therapeutically accessible target whose engagement can produce reproducible clinical benefit.

## 7 Conclusions and future perspectives

CLDN18.2 has become an important membrane target in precision oncology, and its success in gastric/GEJ adenocarcinoma provides proof of concept for cross-tumor development. In CRC, however, pathology-based studies show a more restricted pattern: CLDN18.2 positivity is rare in unselected conventional adenocarcinoma but enriched in CMA, mucin-rich CRC, and tumors with gastric-type or foregut-like differentiation. At present, CLDN18.2 should not be considered an established therapeutic target, prognostic biomarker, or predictive biomarker in CMA. The most rigorous position is that it is a candidate stratification biomarker linked to lineage differentiation and target accessibility. Its clinical utility will ultimately depend on whether CLDN18.2 can be reproducibly identified as a therapeutically accessible biomarker and prospectively linked to meaningful clinical benefit in selected patients with CMA. This measured framing should help future studies avoid overextrapolation from gastric cancer while preserving the translational promise of this target in selected CMA populations.

## 8 Search strategy and review scope

For this narrative review, we searched PubMed, Web of Science, and ClinicalTrials.gov from database inception through July 2026 using combinations of the terms “CLDN18.2”, “claudin 18.2”, “colorectal mucinous adenocarcinoma”, “mucinous colorectal cancer”, “colorectal cancer”, “gastric-lineage differentiation”, “foregut-like differentiation”, “dMMR/MSI-H”, “BRAF V600E”, “CIMP”, “immunohistochemistry”, “zolbetuximab”, “antibody-drug conjugate”, “CAR-T”, and “T-cell engager”. We prioritized peer-reviewed pathology cohorts, translational studies, clinical trials, guidelines, and high-quality reviews directly relevant to CLDN18.2 biology, diagnostic assessment, or therapeutic development. Studies with limited relevance to CLDN18.2 expression, diagnostic methodology, biological mechanisms, or therapeutic translation were not prioritized. Because CMA-specific clinical data remain sparse, evidence was interpreted according to its disease context. Studies directly evaluating CMA or CRC were considered direct disease-relevant evidence, whereas data from gastric/GEJ adenocarcinoma and other CLDN18.2-expressing gastrointestinal tumors were used only as extrapolative evidence to inform biological plausibility, therapeutic mechanisms, and translational hypotheses.
